# Hepatitis B vaccine among healthcare workers: factors associated with the dimensions of the Health Belief Model

**DOI:** 10.1590/1980-549720240036

**Published:** 2024-07-01

**Authors:** Yvanilson Costas Farias, Fernanda de Oliveira Souza, Deisy Vital dos Santos, Margarete Costa Heliotério, Paloma de Sousa Pinho, Tânia Maria de Araújo

**Affiliations:** I Universidade Federal do Recôncavo da Bahia, Health, Education and Work Center – Santo Antônio de Jesus (BA), Brazil.; II Universidade Estadual de Feira de Santana, Epidemiology Center – Feira de Santana (BA), Brazil.

**Keywords:** Health belief model, Hepatitis B vaccines, Health personnel, Attitude of health personnel, Vaccination, Cross-sectional studies

## Abstract

**Objective:**

To investigate the association between the dimensions of the Health Belief Model (HBM) and complete vaccination for hepatitis B among healthcare workers (HCW).

**Methods:**

Cross-sectional epidemiological study with HCW in Primary Health and Medium Complexity Care. Univariate and bivariate analyses were performed to test the association between the outcome variable (complete vaccination for hepatitis B based on self-report) and the variables of the HBM dimensions. Prevalence ratio (PR) and its respective 95% confidence intervals (95%CI) were calculated.

**Results:**

453 HCW participated. The prevalence of complete vaccination for hepatitis B was 56.9%. In the final analysis model, the following variables were associated with complete vaccination for hepatitis B: chances of having hepatitis B (PR=1.73) – related to the susceptibility dimension; disease severity (PR=0.74) – related to severity; reduced risk of absenteeism (PR=1.29) – related to benefits; not spending time to get vaccinated (PR=1.41) and not worrying about Events Supposedly Attributable to Vaccination or Immunization (PR=1.43) – related to barriers.

**Conclusions:**

The completeness of the hepatitis B vaccination schedule, reported by the investigated HCW, reveals the prevalence is below the target established by the Ministry of Health, which follows the national scenario of low coverage presented for other age groups. Understanding the risk perception and severity of hepatitis B can contribute to increasing the prevalence of vaccination for this infection.

## INTRODUCTION

Hepatitis B is a viral infection transmitted vertically, parenterally and sexually; however, it is vaccine-preventable^1^. The World Health Organization (WHO) estimates that 3.5% of the world population lives with the infection by the hepatitis B virus (HBV)^2^. In Brazil, between 2000 and 2022, 276,646 cases of hepatitis B were diagnosed. In 2022, the detection rate was 4.3 cases/100 thousand inhabitants^3^.

Vaccination for hepatitis B is part of the immunization schedule in all life cycles, including among adults (20 to 59 years of age), available universally and for free in the Unified Health System (SUS). Brazilian health workers (HW) are a target-group for vaccination in the National Immunization Program (PNI), for the health and safety of the workers and the assisted populations^1^. Immunization is also recommended by Regulatory Standard 32 – Safety and Health in Health Services (NR 32)^4^. Among HWs, the prevalence of global infection by HBV ranges from 4.8 to 11.1%, and can be even three times higher in comparison to the general population^5^.

Despite the indispensability of completing the vaccination schedule for hepatitis B, which is the most efficient prophylactic measure against the infection^1^, surveys in different parts of the country demonstrated that not all HWs had completed the vaccination schedule nor verified seroconversion through the anti-HBs test. In the city of Montes Claros, in the state of Minas Gerais, 47.5% of the HWs in the Family Health Strategy program did not complete the vaccination schedule for hepatitis B, and only 16.4% were immune to an infection by the HBV^6^. In five cities of the state of Bahia, 40.3% of the HWs did not receive the three doses recommended for the vaccine^7^. Considering the low vaccine coverage, it is believed that HWs are hesitant about being vaccinated against hepatitis B.

Vaccine hesitancy has been classified as one of the ten threats to global health since 2019^8^, being defined as delay in receipt or refusal to vaccinate, despite the availability in vaccination services^9^. There are many reasons for vaccine hesitancy regarding hepatitis B among HWs: doubts about the vaccine’s efficacy and safety; fear of adverse effects; scarce information about the infection’s transmission; lack of risk perception; forgetfulness and difficulties to access the immunizing agent^6,7,10-12^.

In a previous investigation carried out with the current study population, it was observed that 43.1% of HWs hesitated to be vaccinated against hepatitis B; 14.6% were not vaccinated; 2.7% only had one dose; and 5.8% had two doses, whereas the others did not know or did not answer about the number of received doses^13^.

Vaccine hesitancy in Brazil and around the world is investigated by different theoretical models, such as the Health Belief Model (HBM), which aims at understanding the reasons why individuals adopt health-related behaviors or not^14^. When applied to vaccination, the model can help to understand the relationship between behavior and use/acceptance of vaccines.

In the 1950s, social psychologists from the United States developed the HBM to understand the reasons why the population did not attend Public Health actions^15^. In HBM, there are four central dimensions: susceptibility, which is the belief related to the chances of experimenting a risk or contracting a disease or condition; severity, belief about the severity of a condition and its aftereffects if untreated; benefits, belief in the efficacy of an action recommended to mitigate risk or severity of the impact; and barriers, beliefs about the tangible and psychological costs of a recommended action^11-16^. Besides, there are dimensions of stimuli for action and self-efficacy, which can also influence adherence to health actions^17^.

In the vaccination context, the HBM was used to investigate adherence to vaccines against COVID-19^18^ and Influenza^19^. However, the analysis of adherence to vaccination Against hepatitis B, based on this model, is incipient, especially regarding HWs: one of the references found in the literature does not use the HBM itself, despite working with the term “beliefs”^20^, and another survey assesses the prevention of accidents with needles using the HBM^21^.

This study aimed at investigating the association between the dimensions of HBM and complete vaccination for hepatitis B and HWs.

## METHODS

A cross-sectional epidemiological study carried out from June, 2019, to January, 2020, conducted with a randomly selected representative sample, including HWs in Primary Health Care (PHC) and Medium Complexity (MC) in the city of Santo Antônio de Jesus, in Recôncavo of Bahia. This study is part of the multicenter project “Surveillance and Monitoring of Infectious Diseases among Male and Female Workers of the Health Sector”.

The study population selection followed the procedures: previous survey of the population of reference and the health services network for sampling calculation through nominal listing; definition of parameters for sampling calculation; stratification of the population by level of care and occupational groups applicable to the sample; raffle.

Since this is a comprehensive study, the sample calculation was based on an estimation of prevalence rates for different health outcomes; therefore, the obtained sample was larger than the estimated for the analysis of the vaccine situation. For sample calculation, we considered the total population of HWs connected to the services (622), prevalence of complete vaccination for hepatitis B of 79.2%, 3% error and 95% confidence interval. In order to investigate the outcome of interest, a representative sample of 332 HWs was estimated.

Considering possible refusals and loss of information, and as a strategy to guarantee the reach of the sample size for the considered strata, we defined an extra list per random procedure for potential replacement of workers who were not found or those who refused to participate. The extra list was limited to 20% of the original list. In cases of absence or refusal of the selected worker, or after three attempts of conducting an interview, the interviewer was advised to replace this person with another worker of the same level of service care, same occupation and same gender as the person who was initially selected.

The inclusion criteria were: working in PHC or MC services, minimum age of 18 years, and having been working in municipal health services for more than 6 months. Workers on leave or on vacation were excluded.

The occupational groups were divided in four categories, considering the proximity of work characteristics and occupational risks: care (laboratory, nursing and oral health technicians; nurses; physicians; dentists; nutritionists; psychologists; pharmacists; social workers; occupational therapists and physical therapists); health agents (Community Health Agents and Endemic Disease Control Agents); administration (employees in administrative sectors and receptionists; pharmacy assistants; coordinators; assistant managers for special projects and district coordinators); and operational support (general services’ employees; security guards; musicians; cafeteria workers; drivers and; typists; medical regulation assistant technicians; radio operators; sanitary inspectors and fiscals).

Data collection was carried out through a structured questionnaire, elaborated from literature. The “outcome” variable was the self-report of hepatitis B vaccination, obtained through the following questions: “Have you been vaccinated against hepatitis B?” and “If affirmative, did you receive: 1 dose, 2 doses, 3 doses, or you don’t know?. The complete vaccination for hepatitis B was considered for HWs show answered, respectively, “Yes” and “3 doses”, according to the vaccine schedule established for adults by the PNI^1^.

The HBM variables were based on an adapted^17^, unvalidated instrument, since this is an unpublished study. The variables were divided in the following dimensions: susceptibility (perception of risk of contracting hepatitis B and labor exposure to the infection); severity (concern about contracting hepatitis B, about the consequences from acquiring the infection and the severity of the disease); benefits (perception of individual and family protection regarding the vaccine for hepatitis B, reduced risk of missing work days and personal gain); stimuli for action (motivations to adhere to hepatitis B vaccination) and barriers (doubts about hepatitis B vaccination); fear of Events Supposedly Attributable to Vaccination or Immunization [ESAVI] or pain; perception of interference in the routine, associated with spending time or difficulty to commute).

Statistical analyses were conducted using the statistical software Statistical Package for the Social Sciences (SPSS) and STATA: Software for Statistics and Data Science. Initially, there was a univariate analysis, with characterization of the population according to descriptive variables and prevalence (self-report) of complete vaccination for hepatitis B, estimated absolute and relative frequencies, based on SPSS. In SPSS, the bivariate analysis was performed to test the association between the dependent variable (complete vaccination for hepatitis B) and the variables of the HBM dimensions. Prevalent ratio (PR) and its respective 95% confidence intervals (95%CI) were calculated.

In STATA, there was a multivariate analysis that described the simultaneous effect of the variables of interest in the completeness of the vaccination schedule. For that, we selected the variables based on a literature review, verifying the model assumptions and pre-selecting the variables, considering p≤0.20 in the bivariate analysis. The logistic regression model, with odds ratio (OR) correction for PR, was performed based on the Poisson Regression with robust variance, as well as its respective confidence intervals. To evaluate the statistical significance measure, we used the Pearson χ^2^ test, adopting p<0.05.

## RESULTS

Four hundred fifty-three HWs attended the study, of whom 421 had complete information for the variables of interest related to hepatitis B, according to the sample, which represents a global response proportion of 93%. About the completeness of the vaccination schedule for hepatitis B, 56.9% of HWs reported complete vaccination. The group of care HWs obtained the highest prevalence of complete vaccination for hepatitis B (76.6%), followed by health agents (52%), administrative employees (46.3%) and operational support (37.8%).

The study population is mostly female (82.8%) and black (83.6%); aged between 21 and 49 years (76%); with a partner (60.9%) and children (73.9%). About schooling, 61.8% of HWs had incomplete higher education. About occupation, PHC workers were prevalent (77.7%), with effective contract (72.4%), working as health agents (39.4%), followed by care (30.9%), administrative (21.4%) and operational support workers (8.3%).

In bivariate analysis, considering the susceptibility dimension, only the perception about the high chances of contracting hepatitis B was associated with complete vaccination for hepatitis B (PR=1.39; 95%CI 1.19-1.62) (Table 1). There was no association between the factors of the severity dimension and the completeness of the vaccination schedule for hepatitis B (Table 2). About the benefits dimension, three variables were associated with the completeness of the vaccination schedule for hepatitis B by HWs: the perception that vaccination for hepatitis B will protect the people who live with the worker from contracting the infection (PR=1.22; 95%CI 1.04–1.43); the understanding that vaccination for hepatitis B can reduce the chances of missing work (PR=1.22; 95%CI 1.03–1.46); and the understanding about personal gain provided by vaccination for hepatitis B (PR=1.88; 95%CI 1.14–3.09) (Table 3). There was no association between the factors of the stimuli dimension for action and completeness of the vaccination schedule for hepatitis B (Table 3).

**Table 1:** Prevalence of complete vaccination for hepatitis B (three doses) according to the susceptibility dimension in the Health Belief Model among Primary Health Care and Medium Complexity. Santo Antônio de Jesus (BA), 2019–2020.

Variables*	n (%)	Prevalence of complete vaccination n (%)	PR	95%CI	p-value
Working with a loto f people everyday increases my chances of contracting hepatitis B (n=450)					
Yes	154 (34.2)	93 (60.4)	1.10	0.93–1.30	0.250
No	296 (65.8)	162 (54.7)	-	-	-
I have high chances of contracting hepatitis B (n=451)					
Yes	136 (30.2)	96 (70.6)	**1.39**	**1.19–1.62**	**<0.001**
No	315 (69.8)	160 (50.8)	-	-	-
I think my chances of contracting hepatitis B in the near future are high (n=446)					
Yes	48 (10.8)	28 (58.3)	1.03	0.80–1.33	0.812
No	398 (89.2)	225 (56.5)	-	-	-
I really worry about the possibility of contracting hepatitis B (n=448)					
Yes	216 (48.2)	113 (60.6)	1.13	0.96–1.33	0.124
No	232 (51.8)	124 (53.4)	-	-	-

PR: prevalence ratio; 95%CI: 95% confidence interval. *The subtotals are different due to missing data (losses and refusals). Bold means p-value<0.05.

**Table 2:** Prevalence of complete vaccination for hepatitis B (three doses) according to the severity dimension in the Health Belief Model among Primary Health Care and Medium Complexity. Santo Antônio de Jesus (BA), 2019–2020.

Variables*	n (%)	Prevalence of complete vaccination n (%)	PR	95%CI	p-value
It is scary to think I can contract hepatitis B (n=446)					
Yes	252 (56.5)	148 (58.7)	1.08	0.91–1.28	0.330
No	194 (43.5)	105 (54.1)	-	-	-
If I contracted hepatitis B, it could affect my job (n=447)					
Yes	168 (37.6)	99 (58.9)	1.06	0.90–1.25	0.441
No	279 (62.4)	154 (55.2)	-	-	-
If I contracted hepatitis B, it could impact my family (n=446					
Yes	255 (57.2)	150 (58.8)	1.10	0.93–1.30	0.253
No	191 (42.8)	102 (53.4)	-	-	-
Having hepatitis B would make daily activities more difficult (n=448)					
Yes	265 (59.2)	148 (55.8)	0.95	0.81–1.12	0.582
No	183 (40.8)	107 (58.5)	-	-	-
If I contracted hepatitis B, it would be more severe than other diseases (n=450)					
Yes	86 (19.1)	43 (50.0)	0.85	0.68–1.07	0.151
No	364 (80.9)	213 (58.5)	-	-	-
Hepatitis B can be a severe disease (n=452)					
Yes	414 (91.6)	239 (57.7)	1.15	0.83–1.60	0.357
No	38 (8.4)	19 (50.0)	-	-	-

PR: prevalence ratio; 95%CI: 95% confidence interval. * The subtotals are different due to missing data (losses and refusals).

**Table 3:** Prevalence of complete vaccination for hepatitis B (three doses) according to the benefits and encouragement for action dimension in the Health Belief Model among Primary Health Care and Medium Complexity. Santo Antônio de Jesus (BA), 2019–2020.

Variables*	n (%)	Prevalence of complete vaccination n (%)	PR	95%CI	p-value
Being vaccinated against hepatitis B will prevent me from contracting hepatitis B (n=453)					
Yes	284 (62.7)	170 (59.9)	1.15	0.96–1.36	0.105
No	169 (37.3)	88 (52.1)	-	-	-
Being vaccinated against hepatitis B will protect the people who live with me from contracting hepatitis B (n=451)					
Yes	201 (44.6)	127 (63.2)	**1.22**	**1.04–1.43**	**0.014**
No	250 (55.4)	129 (51.6)	-	-	-
Being vaccinated against hepatitis B can reduce my chances of missing work (n=448)					
Yes	264 (58.9)	162 (61.4)	**1.22**	**1.03–1.46**	**0.017**
No	184 (41.1)	92 (50.0)	-	-	-
I have a lot to gain by vaccinating against hepatitis B (n=450)					
Yes	415 (92.2)	246 (59.3)	**1.88**	**1.14–3.09**	**0.001**
No	35 (7.8)	11 (31.4)	-	-	-
I would not be afraid of contracting hepatitis B if I were vaccinated against hepatitis B (n=449)					
Yes	89 (51.2)	141 (61.3)	1.15	0.98–1.36	0.074
No	219 (48.8)	116 (53.0)	-	-	-
Working in health services is a reason to receive a hepatitis B vaccine (n=450)					
Yes	399 (88.7)	231 (57.9)	1.18	0.88–1.58	0.228
No	51 (11.3)	25 (49.0)	-	-	-
I was vaccinated against hepatitis B because a friend or relative encouraged me to do so (n=443)					
Yes	55 (12.4)	30 (54.5)	0.94	0.73–1.22	0.681
No	388 (87.6)	223 (57.5)	-	-	-
I was vaccinated against hepatitis B because there was a campaign at my work site (n=448)					
Yes	172 (38.4)	102 (59.3)	1.06	0.90–1.25	0.466
No	276 (61.6)	154 (55.8)	-	-	-
I was vaccinated against hepatitis B after knowing about the benefits of the vaccine in the communication means (n=447)					
Yes	224 (50.1)	131 (58.5)	1.06	0.90–1.24	0.478
No	223 (49.9)	123 (55.2)	-	-	-
I was vaccinated against hepatitis B because my boss thought it would be important and necessary for the exercise of my occupation (n=447)					
Yes	104 (23.3)	64 (61.5)	1.09	0.91–1.31	0.315
No	343 (76.7)	192 (56.0)	-	-	-
I was vaccinated against hepatitis B because my coworkers were vaccinated and encouraged me to do the same (n=446)					
Yes	93 (20.9)	51 (54.8)	0.94	0.77–1.16	0.609
No	353 (79.1)	204 (57.8)	-	-	-

PR: prevalence ratio; 95%CI: 95% confidence interval. *The subtotals are different due to missing data (losses and refusals). Bold means p-value<0.05.

About the dimension of barriers for vaccination, five factors were associated with the report of complete vaccination for hepatitis B by HWs: those who would not be in doubt about getting a vaccine for hepatitis B, even though the news claims the vaccine can cause health problems (PR=1.46; 95%CI 1.15–1.85); those who acknowledged that it would not be a waste of time to get vaccinated (PR=1.79; 95%CI 1.25–2.56); those who did not believe the vaccine would interfere in their daily lives (PR=1.61; 95%CI 1.14–2.26); those who disagreed that getting a vaccine would be difficult for requiring them to commute (PR=1.56; 95%CI 1.04–2.35); and those who do not worry about a reaction cause by the hepatitis B vaccine (PR=1.61; 95%CI 1.24–2.09) (Table 4).

**Table 4:** Prevalence of complete vaccination for hepatitis B (three doses) according to the barriers dimension in the Health Belief Model among Primary Health Care and Medium Complexity. Santo Antônio de Jesus (BA), 2019–2020.

Variables*	n (%)	Prevalence of complete vaccination n (%)	PR	95%CI	p-value
Some news that states vaccines can cause health problems makes me have doubts about getting a vaccine (n=447)					
No	340 (76.1)	209 (61.5)	**1.46**	**1.15–1.85**	**<0.001**
Yes	107 (23.9)	45 (42.1)	-	-	-
Being vaccinated against hepatitis B can be painful (n=452)					
No	299 (66.2)	176 (58.9)	1.11	0.93–1.13	0.229
Yes	153 (33.8)	72 (52.9)	-	-	-
Being vaccinated against hepatitis B would take too much of my time, because three doses are necessary (n=449)					
No	387 (86.2)	235 (60.7)	**1.79**	**1.25–2.56**	**<0.001**
Yes	62 (13.8)	21 (33.9)	-	-	-
Being vaccinated against hepatitis B can interfere in my daily activities (n=448)					
No	389 (86.8)	234 (60.1)	**1.61**	**1.14–2.26**	**0.001**
Yes	59 (13.2)	22 (37.3)	-	-	-
There are risks associated with the hepatitis B vaccine (n=449)					
Não	327 (72.8)	195 (59.6)	1.19	0.97–1.45	0.067
Sim	122 (27.2)	61 (50.0)	-	-	-
It is hard for me to receive a vaccine against hepatitis B because it would require commuting (n=451)					
No	411 (91.1)	241 (58.6)	**1.56**	**1.04–2.35**	**0.010**
Yes	40 (8.9)	15 (37.5)	-	-	-
I am concerned about having a reaction to the hepatitis B vaccine (n=451)					
No	352 (78.0)	218 (61.9)	**1.61**	**1.24–2.09**	**<0.001**
Yes	99 (22.0)	134 (35.4)	-	-	-
A person can contract hepatitis when getting a vaccine against hepatitis B (n=448)					
No	248 (55.4)	148 (59.7)	1.12	0.95–1.32	0.156
Yes	200 (44.6)	106 (53.0)	-	-	-
People often get sick when getting a hepatitis B vaccine (n=451)					
No	327 (72.5)	194 (59.3)	1.18	0.97–1.44	0.074
Yes	124 (27.5)	62 (50.0)	-	-	-

PR: prevalence ratio; 95%CI: 95% confidence interval. *The subtotals are different due to missing data (losses and refusals). Bold means p-value<0.05.

In the final model of the analysis, the dimensions of susceptibility, severity, benefits and barriers have been associated with complete vaccination for hepatitis B among HWs. The HBM variables associated with complete hepatitis B vaccination, in the multivariate analysis, were: “I have high chances of contracting hepatitis B” (yes) (PR=1.73; 95%CI 1.30–2.29) — susceptibility; “If I contracted hepatitis B, it would be more severe than other diseases” (yes) (PR=0.74; 95%CI 0.58–0.93) — severity; “Being vaccinated against hepatitis B can reduce the chances of missing work” (yes) (PR=1.29; 95%CI 1.05–1.59) — benefits; “Being vaccinated against hepatitis B would take much of my time, because three doses are necessary” (no) (PR=1.41; 95%CI 1.11–1.79) — barriers; “I am concerned about getting a reaction caused by the hepatitis B vaccine” (no) (PR=1.43; 95%CI 1.14–1.80) — barriers (Table 5).

**Table 5:** Variables obtained in regression analysis final model associated with the prevalence of complete vaccination against hepatitis B (three doses) among Primary Health Care and Medium Complexity workers. Santo Antônio de Jesus (BA), 2019–2020.

Variables	Adjusted PR	95%CI	p-value
I have high chances of contracting hepatitis B (yes) — Susceptibility	1,73	1,30–2,29	<0,001
If I contracted hepatitis B, it would be more severe than other diseases (yes) — Severity	0,74	0,58–0,93	0,012
Being vaccinated against hepatitis B can reduce my chances of missing work (yes) — Benefits	1,29	1,05–1,59	0,014
Being vaccinated against hepatitis B would take too much of my time, because three doses are necessary (no) — Barriers	1,41	1,11–1,79	0,004
I am concerned about having a reaction to the hepatitis B vaccine (no) — Barriers	1,43	1,14–1,80	0,002

PR: prevalence ratio; 95%CI: 95% confidence interval.

It is important to mention that the loss of 7% of the workers (n=32) in this sample, due to the lack of information for the variables related to hepatitis B, was similar to the group of participants regarding the characteristics of gender and age. In the group of losses, 78.1% of HWs were female, and 75% were aged between 21 and 49 years; thus, the study susceptibility is reduced to a possible selection bias due to non-response.

## DISCUSSION

The prevalence of complete vaccination for hepatitis B (self-report) among HWs in this study (56.9%) was lower than that observed in a survey conducted in Bahia, in 2018, with HWs from PHC and MC (59.7%)^7^, and in a previous survey carried out with the same group of HWs in the city of Santo Antônio de Jesus in 2015 (59.9%)^22^. However, the prevalence of complete vaccination for hepatitis B was higher than that found in a study with HWs from the Family Health Strategy in Montes Claros, in the state of Minas Gerais, in 2015 (52.5%)^6^.

The following were associated with higher prevalence of complete vaccination for hepatitis B: risk perception for the disease; severity in relation to other diseases; perception that the vaccination could reduce absenteeism; no concern about the time spent to vaccinate and the occurrence of ESAVI.

Vaccination for hepatitis B is an important prevention strategy, because 15 to 40% of infected people develop liver failure, liver cirrhosis, and hepatocellular carcinoma, compromising the productive lives of the affected population^11^. An investigation showed that, in 2005, the mean costs per patient, in the public sector, ranged between R$ 1,243,17 and R$ 22,022,61, depending on the complication caused by hepatitis B^23^. After approximately 20 years, it is inferred that these costs increased considerably.

The perceived susceptibility refers to the subjective risks of acquiring a specific condition^16^. Therefore, the perception of susceptibility ranges according to the level of risk to which an individual perceives oneself to be exposed or not, which is a determinant condition for vaccination adherence in general^8^. About vaccination for hepatitis B, there is an association between perception of susceptibility and more adherence to vaccination, in order to prevent the infection among HWs^7,22,24^.

Possibly, this association is owed to occupational exposure, since HWs have up to three times more chances of acquiring a hepatitis B infection in comparison to the general population^5^. It is estimated that the risk of contracting hepatitis B after a needlestick injury can reach 30%. Globally, occupational exposure is responsible for approximately 40% of the hepatitis B and C cases, and for about 66 thousand hepatitis B infections among HWs^25^. In this study, HWs who reported high chances of contracting hepatitis B presented 70% more chances of being vaccinated in comparison to HWs without such a risk perception.

In 2012, an investigation with HWs from the municipal sector of Belo Horizonte, and a survey with HWs from PMC and MC in Santo Antônio de Jesus, in Bahia, observed that workers in the administrative and general services sectors do not see themselves at risk in their occupations, due to the less frequent contact with the service user. However, the risk of contact with biological material or contaminated surfaces is not null; therefore, vaccination remains as the most efficient prophylactic and safe measure to prevent hepatitis B infection.

Perceived severity refers to the possibility of the disease to cause death, reduce physical or mental functioning for long periods, or disable permanently^14^. In this study, HWs who do not understand the severity of hepatitis B obtained higher prevalence of complete vaccination for the infection. This scenario is related to the fact that most of the study participants do not have specific graduation in health courses (only 30.9% were care workers), in which the disciplines related to infections provide more understanding about the severity of hepatitis B.

In a study conducted with nurses in two hospitals from the countryside of the State of São Paulo, in 2012, 77.3% of the participants reported fear of acquiring a severe infection, such as hepatitis B and C, and 92.5% considered themselves to be susceptible to these infections^20^. Hepatitis B is a severe infection, whose repercussions burden the health system^23^.

The benefits dimension is characterized as the belief in the efficacy of the recommended action to reduce risk or severity of the impact^17^. In this study, a higher prevalence of complete vaccination was associated with the reduction of chances of missing work. IN 2012, a survey carried out in two hospitals in São Paulo, whose participants were nurses, obtained a similar result: 100% of the interviewed workers stated that the main benefit of getting a vaccine against hepatitis B is the prevent contracting the infection and, consequently, mitigating the occurrence of days missed at work^20^.

Despite the validity of the benefit perceived for the HW, it is inferred that its thought is based on financial optics, which prevents absenteeism, so there is no risk of unemployment. So, the decision of these HWs to get vaccinated against hepatitis B was not related to their health, but instead, it was associated with an economic matter.

The barriers in the HBM are effective actions to reduce the threat of disease or condition; however, they can be considered as inconvenient, expensive, unenjoyable or painful^15^. In this study, an interval of up to six months between vaccine doses for hepatitis B was not a detrimental factor for the completeness of the vaccination schedule for the infection. An investigation conducted among HWs from Chinese hospitals showed that an interval between doses can lead to loss of opportunity for vaccination, because of the HWs who did not receive the three doses of the vaccine, 40% claimed they were very busy to receive the following doses^12^.

Besides, HWs from PMC were prevalent in this study (77.7%), and this factor can promote hepatitis B vaccines, since it is part of the routine of services in this level of health care, as observed in a previous study with the same population in this city^13^. Whereas HWs from MC perform activities that are little related with vaccination and are far from the vaccine room, PHC workers receive vaccines at their work site, and there is encouragement to update their vaccination cards.

HWs who did not report fear about the occurrence of ESAVI reached higher prevalence of hepatitis B vaccines, with 43% more chances of getting a vaccine in comparison to HWs who reported concern about ESAVI. Probably, this scenario is related to the understanding about the low reactogenicity of the hepatitis B vaccine: ESAVI are often mild and temporary; severe ones are rare^1^ and not a barrier for vaccination. In 2011, a Brazilian cross-sectional study carried out in the state of Minas Gerais showed only 2.1% of incidence of ESAVI in hepatitis B vaccines, being the fourth vaccine with the lowest incidence^26^.

In 2019, in Saudi Arabia, a survey with care workers observed that 31.2% of HWs did not complete the vaccination schedule for hepatitis B and believed that the vaccine was not safe, or had doubts about its safety, whereas only 8% of the HWs who received the three doses had the same belief^11^.

Among the study limitations, there is social desirability and recall bias, especially when HWs were asked about the hepatitis B vaccination^27^. In order to minimize the bias, a copy of HWs vaccination cards was requested, which is an ideal and recommended strategy to assess the outcome of interest^28^. However, it was observed that these HWs relatively know their vaccination status, so a self-report for hepatitis B vaccines was considered for analysis.

Despite the aforementioned limitations, this study stands out for including not only health professionals, but also other occupations in the health field. So, even though this is a local study, it is likely to represent a problem that exists in other parts of the country.

This study shows that the vaccination rate for hepatitis B in this population is lower than expected, and indicates how the perception of risk and severity of the disease can enhance the acceptance of vaccines and the completeness of vaccination schedules for hepatitis B. Therefore, educational activities in services for HWs should include dialogue as a strategy to encourage vaccination through approaches that raise awareness about expected post-vaccination adverse effects, and that the possibility of becoming ill due to an immunopreventable infection, especially hepatitis B, can lead not only to missed work days, but also to hospitalization and death. Finally, regardless of the training and performed role, HWs should assimilate the main forms of infection transmission and recognize vaccination as an indispensable prevention tool.
