# Supplementary material for: Hepatitis B vaccine among healthcare workers: factors associated with the dimensions of the Health Belief Model
Source: Rev Bras Epidemiol. 2024 Jul 1;27:e240036. doi: 10.1590/1980-549720240036 (PMC11221482; doi:10.1590/1980-549720240036)
Supplement: Translation

# Translation: Vacina para hepatite B entre trabalhadores da saúde: fatores associados às dimensões do Modelo de Crenças em Saúde

**DOI:** 10.1590/1980-549720240036.2

**Authors:** Yvanilson Costas Farias, Fernanda de Oliveira Souza, Deisy Vital dos Santos, Margarete Costa Heliotério, Paloma de Sousa Pinho, Tânia Maria de Araújo

**Affiliations:** I Universidade Federal do Recôncavo da Bahia, Núcleo de Saúde, Educação e Trabalho – Santo Antônio de Jesus (BA), Brasil.; II Universidade Estadual de Feira de Santana, Núcleo de Epidemiologia – Feira de Santana (BA), Brasil.

**Keywords:** Modelo de crenças de saúde, Vacinas contra hepatite B, Pessoal de saúde, Atitude do pessoal de saúde, Vacinação, Estudos transversais

## Abstract

**Objetivo:**

Investigar a associação entre as dimensões do Modelo de Crenças em Saúde (MCS) e a vacinação completa para hepatite B entre trabalhadores da saúde (TS).

**Métodos:**

Estudo epidemiológico de corte transversal com TS da Atenção Primária à Saúde e Média Complexidade. Realizaram-se análises uni e bivariada a fim de testar a associação entre a variável desfecho (vacinação completa para hepatite B a partir do autorrelato) e as variáveis das dimensões do MCS. Foram calculadas razões de prevalência (RP) e seus respectivos intervalos de confiança de 95% (IC95%).

**Resultados:**

Participaram do estudo 453 TS. A prevalência de vacinação completa para hepatite B foi de 56,9%. No modelo final de análise, estiveram associadas à vacinação completa para hepatite B as variáveis: chance de pegar hepatite B (RP=1,73) – relativa à dimensão de suscetibilidade; gravidade da doença (RP=0,74) – relativa à dimensão de gravidade; diminuição do risco de absenteísmo (RP=1,29) – relativa à dimensão de benefícios; não dispêndio de tempo para se vacinar (RP=1,41) e não preocupação acerca de eventos atribuíveis à vacinação ou imunização (RP=1,43) – relativas à dimensão de barreiras.

**Conclusão:**

A completude do esquema vacinal para hepatite B, referida pelos TS investigados, revela uma prevalência abaixo da meta estabelecida pelo Ministério da Saúde, a qual acompanha o cenário nacional de baixas coberturas apresentado para outras faixas etárias. A compreensão sobre percepção de risco e gravidade da hepatite B pode contribuir para o aumento da prevalência de vacinação para essa infecção.

## INTRODUÇÃO

A hepatite B é uma infecção viral de transmissão vertical, parenteral e sexual, todavia, imunoprevenível^1^. A Organização Mundial da Saúde (OMS) estima que 3,5% da população mundial vive com a infecção pelo vírus da hepatite B (VHB)^2^. No Brasil, entre 2000 e 2022, diagnosticaram-se 276.646 casos de hepatite B. Em 2022, a taxa de detecção foi de 4,3 casos/100 mil habitantes^3^.

A vacinação para hepatite B está no calendário de vacinação em todos os ciclos de vida, incluindo indivíduos adultos (20 a 59 anos), disponível de forma universal e gratuita no Sistema Único de Saúde (SUS). Trabalhadores da saúde (TS) brasileiros são um grupo-alvo de vacinação do Programa Nacional de Imunização (PNI), para saúde e segurança do próprio trabalhador e das populações assistidas^1^. A vacinação também está recomendada pela Norma Regulamentadora 32 – Segurança e Saúde no Trabalho em Serviços de Saúde (NR 32)^4^. Em TS, a prevalência da infecção global pelo VHB varia de 4,8 a 11,1% e pode ser até três vezes maior comparada à população geral^5^.

Apesar da imprescindibilidade da completude do esquema vacinal para hepatite B, medida profilática mais eficaz contra a infecção^1^, inquéritos em distintas partes do país demonstraram que nem todos os TS completaram o esquema vacinal nem verificaram a soroconversão por meio do exame Anti-HBs. No município de Montes Claros, no estado de Minas Gerais, 47,5% dos TS da Estratégia da Saúde da Família não completaram o esquema vacinal para hepatite B e apenas 16,4% estavam imunes à infecção pelo VHB^6^. Em cinco municípios do estado da Bahia, 40,3% dos TS não receberam as três doses recomendas da vacina^7^. Considerando a baixa cobertura vacinal, acredita-se que TS estejam hesitantes para se vacinar contra a hepatite B.

A hesitação vacinal foi classificada como uma das dez ameaças à saúde global desde 2019^8^, sendo definida como atraso no recebimento ou recusa da vacinação, apesar da disponibilidade nos serviços de vacinação^9^. Diversas são as razões para hesitação vacinal para hepatite B entre TS: dúvidas sobre eficácia e segurança da vacina; negligência; receio de reações adversas; informações escassas sobre transmissão da infecção; ausência de percepção de risco; esquecimento e dificuldade de acesso ao imunizante^6,7,10-12^.

Em investigação anterior, realizada com a atual população do estudo, constatou-se que 43,1% dos TS hesitaram em vacinar-se contra hepatite B, 14,6% não se vacinaram, 2,7% receberam apenas uma dose e 5,8% receberam duas, enquanto os demais não sabiam ou não responderam o número de doses recebidas^13^.

A hesitação vacinal no Brasil e no mundo é investigada por distintos modelos teóricos, como o Modelo de Crenças em Saúde (MCS), que visa compreender os motivos pelos quais indivíduos adotam ou não comportamentos relacionados à saúde^14^. Aplicando-se à vacinação, o modelo pode auxiliar na compreensão da relação entre comportamento e utilização/aceitação de vacinas.

Na década de 1950, psicólogos sociais estadunidenses desenvolveram o MCS a fim de compreender os motivos pelos quais a população não aderia a ações de Saúde Pública^15^. No MCS, quatro dimensões são centrais: suscetibilidade, crença relacionada às chances de experimentar um risco ou contrair determinada condição ou doença; gravidade, crença acerca da severidade de uma condição e suas sequelas se não tratá-la; benefícios, crença na eficácia de uma ação recomendada para mitigar risco ou gravidade do impacto; e barreiras, crenças sobre os custos tangíveis e psicológicos de uma ação recomendada^11-16^. Ademais, há as dimensões de estímulos para ação e autoeficácia, que também podem influenciar na adesão a ações de saúde^17^.

No contexto da vacinação, utilizou-se o MCS para a investigação da adesão às vacinas para COVID-19^18^ e Influenza^19^. Entretanto, a análise da adesão à vacinação para hepatite B, a partir desse modelo, é incipiente, principalmente em relação aos TS: uma das literaturas encontradas não utiliza o MCS em si, apesar de trabalhar com o termo “crenças”^20^ e outro inquérito avalia a prevenção dos acidentes com agulhas a partir do MCS^21^.

Este estudo objetivou investigar a associação entre dimensões do MCS e vacinação completa para hepatite B entre TS.

## MÉTODOS

Estudo epidemiológico transversal realizado no período de junho de 2019 a janeiro de 2020, conduzido com amostra representativa, aleatoriamente selecionada, de TS de Atenção Primária à Saúde (APS) e Média Complexidade (MC) do município de Santo Antônio de Jesus no Recôncavo da Bahia. Este estudo integra o projeto multicêntrico “Vigilância e Monitoramento de Doenças Infecciosas entre Trabalhadores e Trabalhadoras do Setor Saúde”.

A seleção da população do estudo seguiu os procedimentos: levantamento prévio da população de referência e da rede de serviços de saúde para cálculo amostral por meio de listagem nominal; definição de parâmetros para cálculo amostral; estratificação da população por nível de atenção e grupos ocupacionais aplicáveis à amostra; sorteio.

Por tratar-se de uma pesquisa abrangente, realizou-se cálculo amostral como estimativa de prevalências para distintos desfechos em saúde, assim, o tamanho da amostra obtida foi superior ao estimado para a análise da situação vacinal. Para o cálculo amostral, considerou-se a população total de TS vinculados aos serviços (622), prevalência de vacinação completa para hepatite B de 79,2%, erro de 3% e nível de confiança de 95%. A fim de investigar o desfecho de interesse, estimou-se amostra representativa de 332 TS.

Considerando eventuais recusas e perda de informações e como estratégia para garantir o alcance do tamanho amostral para estratos considerados, definiu-se uma lista extra (reserva) por procedimento aleatório para eventual substituição de trabalhadores não encontrados ou recusas. A lista extra limitou-se a 20% da listagem original. Em casos de ausência ou recusa do trabalhador sorteado ou após três tentativas de realização da entrevista, orientou-se o entrevistador a substitui-lo por outro trabalhador do mesmo nível de atenção do serviço, da mesma ocupação e do mesmo sexo daquele trabalhador inicialmente sorteado.

Foram critérios de inclusão: trabalhar nos serviços de APS ou MC, idade mínima de 18 anos e estar trabalhando por mais de 6 meses nos serviços de saúde municipais. Excluíram-se TS afastados, de férias ou de licença.

Dividiram-se os grupos ocupacionais em quatro categorias, considerando a proximidade das características do trabalho e de riscos ocupacionais: assistenciais (técnicos de laboratório, de enfermagem e de saúde bucal; enfermeiros; médicos; dentistas; nutricionistas; psicólogos; farmacêuticos; assistentes sociais; terapeutas ocupacionais e fisioterapeutas); agentes de saúde (Agentes Comunitários de Saúde e Agentes de Combate às Endemias); administrativos (funcionários administrativos e recepcionistas; atendentes de farmácia; coordenadores; subgerentes de projetos especiais e coordenadores de distrito) e apoio operacional (funcionários de serviços gerais; seguranças; músicos; merendeiras; motoristas e condutores; digitadores; técnicos auxiliares de regulação médica; operadores de rádio; inspetores e fiscais sanitários).

A coleta de dados foi realizada por questionário estruturado, elaborado a partir da literatura. A variável “desfecho” foi o autorrelato de vacinação para hepatite B, obtido por meio dos seguintes questionamentos: “Já recebeu vacina contra hepatite B?” e “Em caso afirmativo, você recebeu: 1 dose, 2 doses, 3 doses ou não sabe?”. A vacinação completa para hepatite B foi considerada para TS que responderam, respectivamente, “Sim” e “3 doses”, conforme o esquema vacinal do adulto preconizado pelo PNI^1^.

As variáveis do MCS basearam-se em um instrumento adaptado^17^ e não validado, por tratar-se de um estudo inédito. As variáveis foram divididas nas seguintes dimensões: suscetibilidade (percepção de risco de contrair hepatite B e de exposição laboral à infecção); gravidade (preocupação de contrair hepatite B, das consequências advindas da aquisição da infecção e da seriedade da doença); benefícios (percepção de proteção individual e familiar em relação à vacinação para hepatite B, de diminuição do risco de absenteísmo no trabalho e de ganho pessoal); de estímulos para ação (motivações para aderir à vacinação para hepatite B) e barreiras (dúvidas acerca da vacinação para hepatite B; receio de Eventos Supostamente Atribuíveis à Vacinação ou Imunização [ESAVI] ou dor; percepção de interferência no cotidiano, associada a dispêndio de tempo ou dificuldade de deslocamento).

Realizaram-se análises estatísticas pelos programas estatísticos Statistical Package for Social Sciences (SPSS) e STATA: Software for Statistics and Data Science. Inicialmente, realizou-se análise univariada, com caracterização da população de acordo com variáveis descritoras e prevalência (autorrelato) de vacinação completa para hepatite B, estimadas frequências absolutas e relativas, a partir do SPSS. No SPSS, realizou-se análise bivariada a fim de testar a associação entre variável dependente (vacinação completa para hepatite B) e variáveis das dimensões do MCS. Calcularam-se razões de prevalência (RP) e seus respectivos intervalos de confiança de 95% (IC95%).

No STATA, realizou-se análise multivariada, que descreveu o efeito simultâneo das variáveis de interesse na completude do esquema de vacinação. Para tal, realizou-se seleção das variáveis baseada em revisão de literatura, verificação de pressupostos do modelo e pré-seleção das variáveis, considerando p≤0,20 na análise bivariada. O modelo de regressão logística com correção da *odds ratio* (OD) para RP foi realizado a partir da Regressão de Poisson com variância robusta, assim como seus respectivos intervalos de confiança. Para avaliação da medida de significância estatística, utilizou-se teste de χ^2^ de Pearson, adotando p<0,05.

## RESULTADOS

Participaram do estudo 453 TS, dos quais 421 tinham informações completas para as variáveis de interesse relacionadas à hepatite B, conformando a amostra, o que representa uma proporção de resposta global de 93%. Acerca da completude do esquema vacinal para hepatite B, 56,9% dos TS relataram vacinação completa. O grupo dos TS assistenciais obteve a maior prevalência de vacinação completa para hepatite B (76,6%), seguido por agentes de saúde (52%), administrativos (46,3%) e apoio operacional (37,8%).

A população do estudo é predominantemente feminina (82,8%) e negra (83,6%); entre 21 e 49 anos (76%); tem companheiro (60,9%) e filhos (73,9%). Sobre escolaridade, 61,8% dos TS tinham até o ensino superior incompleto. Acerca da ocupação, predominaram trabalhadores da APS (77,7%), com vínculo efetivo (72,4%), na ocupação de agentes de saúde (39,4%), seguidos por assistenciais (30,9%), administrativos (21,4%) e apoio operacional (8,3%).

Na análise bivariada, na dimensão de suscetibilidade, apenas a percepção acerca da grande possibilidade de contrair hepatite B associou-se à vacinação completa para hepatite B (RP=1,39; IC95% 1,19–1,62) (Tabela 1). Não houve associação entre fatores da dimensão de gravidade e completude do esquema vacinal para hepatite B (Tabela 2). Sobre a dimensão de benefícios, três variáveis apresentaram associação à completude do esquema vacinal para hepatite B pelos TS: a percepção de que a vacinação para hepatite B protegerá as pessoas que moram com o trabalhador de contrair a infecção (RP=1,22; IC95% 1,04–1,43); o entendimento de que a vacinação para hepatite B pode reduzir as chances de faltar ao trabalho (RP=1,22; IC95% 1,03–1,46); e a compreensão acerca de um ganho pessoal proporcionado pela vacinação para hepatite B (RP=1,88; IC95% 1,14–3,09) (Tabela 3). Não houve associação entre fatores da dimensão de estímulos para ação e completude do esquema vacinal para hepatite B (Tabela 3).

**Tabela 1:** Prevalência de vacinação completa para hepatite B (três doses) de acordo com a dimensão de suscetibilidade do Modelo de Crenças em Saúde entre trabalhadores da Atenção Primária à Saúde e Média Complexidade. Santo Antônio de Jesus (BA), 2019–2020.

Variáveis*	n (%)	Prevalência de vacinação completa n (%)	RP	IC95%	p-valor
Trabalhar com muitas pessoas todo dia aumenta minhas chances de pegar hepatite B (n=450)					
Sim	154 (34,2)	93 (60,4)	1,10	0,93–1,30	0,250
Não	296 (65,8)	162 (54,7)	-	-	-
Eu tenho grande chance de pegar hepatite B (n=451)					
Sim	136 (30,2)	96 (70,6)	**1,39**	**1,19–1,62**	**<0,001**
Não	315 (69,8)	160 (50,8)	-	-	-
Eu acho que minha chance de pegar hepatite B no futuro próximo é grande (n=446)					
Sim	48 (10,8)	28 (58,3)	1,03	0,80–1,33	0,812
Não	398 (89,2)	225 (56,5)	-	-	-
Eu me preocupo muito com a possibilidade de pegar hepatite B (n=448)					
Sim	216 (48,2)	113 (60,6)	1,13	0,96–1,33	0,124
Não	232 (51,8)	124 (53,4)	-	-	-

RP: razões de prevalência; IC95%: intervalo de confiança de 95%. *Os subtotais diferem devido a dados faltosos (perdas e recusas). Negrito indica valores de p<0.05.

**Tabela 2:** Prevalência de vacinação completa para hepatite B (três doses) de acordo com a dimensão de gravidade do Modelo de Crenças em Saúde entre trabalhadores da Atenção Primária à Saúde e Média Complexidade. Santo Antônio de Jesus (BA), 2019–2020.

Variáveis*	n (%)	Prevalência de vacinação completa n (%)	RP	IC95%	p-valor
Pensar que posso pegar hepatite B me assusta (n=446)					
Sim	252 (56,5)	148 (58,7)	1,08	0,91–1,28	0,330
Não	194 (43,5)	105 (54,1)	-	-	-
Se eu pegasse hepatite B, poderia comprometer meu emprego (n=447)					
Sim	168 (37,6)	99 (58,9)	1,06	0,90–1,25	0,441
Não	279 (62,4)	154 (55,2)	-	-	-
Se eu pegasse hepatite B, isso prejudicaria a minha família (n=446)					
Sim	255 (57,2)	150 (58,8)	1,10	0,93–1,30	0,253
Não	191 (42,8)	102 (53,4)	-	-	-
Estar com hepatite B tornaria as atividades diárias mais difíceis (n=448)					
Sim	265 (59,2)	148 (55,8)	0,95	0,81–1,12	0,582
Não	183 (40,8)	107 (58,5)	-	-	-
Se eu pegasse hepatite B, isso seria mais grave do que outras doenças (n=450)					
Sim	86 (19,1)	43 (50,0)	0,85	0,68–1,07	0,151
Não	364 (80,9)	213 (58,5)	-	-	-
A hepatite B pode ser uma doença grave (n=452)					
Sim	414 (91,6)	239 (57,7)	1,15	0,83–1,60	0,357
Não	38 (8,4)	19 (50,0)	-	-	-

RP: razões de prevalência; IC95%: intervalo de confiança de 95%. *Os subtotais diferem devido a dados faltosos (perdas e recusas).

**Tabela 3:** Prevalência de vacinação completa para hepatite B (três doses) de acordo com a dimensão de benefícios e estímulos para ação do Modelo de Crenças em Saúde entre trabalhadores da Atenção Primária à Saúde e Média Complexidade. Santo Antônio de Jesus (BA), 2019–2020.

Variáveis*	n (%)	Prevalência de vacinação completa n (%)	RP	IC95%	p-valor
Vacinar-me contra hepatite B me impedirá de pegar hepatite B (n=453)					
Sim	284 (62,7)	170 (59,9)	1,15	0,96–1,36	0,105
Não	169 (37,3)	88 (52,1)	-	-	-
Vacinar-me contra hepatite B protegerá as pessoas que moram comigo de pegar hepatite B (n=451)					
Sim	201 (44,6)	127 (63,2)	**1,22**	**1,04–1,43**	**0,014**
Não	250 (55,4)	129 (51,6)	-	-	-
Vacinar-me contra hepatite B pode diminuir as chances de faltar ao trabalho (n=448)					
Sim	264 (58,9)	162 (61,4)	**1,22**	**1,03–1,46**	**0,017**
Não	184 (41,1)	92 (50,0)	-	-	-
Eu tenho muito a ganhar ao me vacinar contra hepatite B (n=450)					
Sim	415 (92,2)	246 (59,3)	**1,88**	**1,14–3,09**	**0,001**
Não	35 (7,8)	11 (31,4)	-	-	-
Eu não teria medo de pegar hepatite B se eu me vacinasse contra hepatite B (n=449)					
Sim	89 (51,2)	141 (61,3)	1,15	0,98–1,36	0,074
Não	219 (48,8)	116 (53,0)	-	-	-
Trabalhar nos serviços de saúde é um motivo para se vacinar contra hepatite B (n=450)					
Sim	399 (88,7)	231 (57,9)	1,18	0,88–1,58	0,228
Não	51 (11,3)	25 (49,0)	-	-	-
Eu me vacinei contra hepatite B porque um amigo ou familiar me estimulou a fazer (n=443)					
Sim	55 (12,4)	30 (54,5)	0,94	0,73–1,22	0,681
Não	388 (87,6)	223 (57,5)	-	-	-
Eu me vacinei contra hepatite B porque foi realizada campanha de vacinação no meu trabalho (n=448)					
Sim	172 (38,4)	102 (59,3)	1,06	0,90–1,25	0,466
Não	276 (61,6)	154 (55,8)	-	-	-
Eu me vacinei contra hepatite B após ouvir informações sobre os benefícios da vacina nos meios de comunicação (n=447)					
Sim	224 (50,1)	131 (58,5)	1,06	0,90–1,24	0,478
Não	223 (49,9)	123 (55,2)	-	-	-
Eu me vacinei contra hepatite B porque meu/minha chefe achou que seria algo importante e necessário para o exercício das minhas atividades laborais (n=447)					
Sim	104 (23,3)	64 (61,5)	1,09	0,91–1,31	0,315
Não	343 (76,7)	192 (56,0)	-	-	-
Eu me vacinei contra hepatite B porque meus colegas de trabalho se vacinaram e me estimularam a fazer vacinação (n=446)					
Sim	93 (20,9)	51 (54,8)	0,94	0,77–1,16	0,609
Não	353 (79,1)	204 (57,8)	-	-	-

RP: razões de prevalência; IC95%: intervalo de confiança de 95%. *Os subtotais diferem devido a dados faltosos (perdas e recusas). Negrito indica valores de p<0.05.

Sobre a dimensão de barreiras para a vacinação, cinco fatores estiveram associados ao relato de vacinação completa para hepatite B pelos TS: aqueles que não ficariam em dúvida sobre a vacinação para hepatite B, apesar de notícias afirmarem que a vacina pode acarretar problemas de saúde (RP=1,46; IC95% 1,15–1,85); aqueles que reconheceram que se vacinar não despenderia tempo (RP=1,79; IC95% 1,25–2,56); aqueles que não acreditam que se vacinar interferiria em sua vida cotidiana (RP=1,61; IC95% 1,14–2,26); aqueles que discordaram que se vacinar seria difícil por exigir deslocamento (RP=1,56; IC95% 1,04–2,35); e aqueles que não se preocupam em relação à ocorrência de reação à vacina para hepatite B (RP=1,61; IC95% 1,24–2,09) (Tabela 4).

**Tabela 4:** Prevalência de vacinação completa para hepatite B (três doses) de acordo com a dimensão de barreiras do Modelo de Crenças em Saúde entre trabalhadores da Atenção Primária à Saúde e Média Complexidade. Santo Antônio de Jesus (BA), 2019–2020.

Variáveis*	n (%)	Prevalência de vacinação completa n (%)	RP	IC95%	p-valor
Algumas notícias que afirmam que a vacina pode trazer problemas de saúde me deixam em dúvida sobre se vacinar (n=447)					
Não	340 (76,1)	209 (61,5)	**1,46**	**1,15–1,85**	**<0,001**
Sim	107 (23,9)	45 (42,1)	-	-	-
Vacinar-me contra hepatite B pode ser doloroso (n=452)					
Não	299 (66,2)	176 (58,9)	1,11	0,93–1,13	0,229
Sim	153 (33,8)	72 (52,9)	-	-	-
Vacinar-me contra hepatite B despenderia muito do meu tempo, pois são necessárias três doses (n=449)					
Não	387 (86,2)	235 (60,7)	**1,79**	**1,25–2,56**	**<0,001**
Sim	62 (13,8)	21 (33,9)	-	-	-
Vacinar-me contra hepatite B pode interferir nas minhas atividades diárias (n=448)					
Não	389 (86,8)	234 (60,1)	**1,61**	**1,14–2,26**	**0,001**
Sim	59 (13,2)	22 (37,3)	-	-	-
Existem riscos associados à vacina da hepatite B (n=449)					
Não	327 (72,8)	195 (59,6)	1,19	0,97–1,45	0,067
Sim	122 (27,2)	61 (50,0)	-	-	-
Para me vacinar contra hepatite B é difícil porque exige deslocamento (n=451)					
Não	411 (91,1)	241 (58,6)	**1,56**	**1,04–2,35**	**0,010**
Sim	40 (8,9)	15 (37,5)	-	-	-
Fico preocupado em ter uma reação à vacina da hepatite B (n=451)					
Não	352 (78,0)	218 (61,9)	**1,61**	**1,24–2,09**	**<0,001**
Sim	99 (22,0)	134 (35,4)	-	-	-
Uma pessoa pode pegar hepatite ao se vacinar contra hepatite B (n=448)					
Não	248 (55,4)	148 (59,7)	1,12	0,95–1,32	0,156
Sim	200 (44,6)	106 (53,0)	-	-	-
Pessoas muitas vezes ficam doentes ao se vacinarem contra hepatite B (n=451)					
Não	327 (72,5)	194 (59,3)	1,18	0,97–1,44	0,074
Sim	124 (27,5)	62 (50,0)	-	-	-

RP: razões de prevalência; IC95%: intervalo de confiança de 95%. *Os subtotais diferem devido a dados faltosos (perdas e recusas). Negrito indica valores de p<0.05.

No modelo final de análise, as dimensões de suscetibilidade, gravidade, benefícios e barreiras estiveram associadas à vacinação completa para hepatite B entre TS. As variáveis do MCS associadas à vacinação completa para hepatite B, na análise multivariada, foram: “Eu tenho grande chance de pegar hepatite B” (sim) (RP=1,73; IC95% 1,30–2,29) — suscetibilidade; “Se eu pegasse hepatite B, isso seria mais grave que outras doenças” (sim) (RP=0,74; IC95% 0,58–0,93) — gravidade; “Vacinar-me contra hepatite B pode diminuir as chances de faltar ao trabalho” (sim) (RP=1,29; IC95% 1,05–1,59) — benefícios; “Vacinar-se contra hepatite B despenderia muito do meu tempo, pois são necessárias três doses” (não) (RP=1,41; IC95% 1,11–1,79) — barreiras; “Fico preocupado em ter uma reação à vacina da hepatite B” (não) (RP=1,43; IC95% 1,14–1,80) — barreiras (Tabela 5).

**Tabela 5:** Variáveis obtidas no modelo final de análise de regressão associadas à prevalência de vacinação completa para hepatite B (três doses) entre trabalhadores da Atenção Primária à Saúde e Média Complexidade. Santo Antônio de Jesus (BA), 2019–2020.

Variáveis	RP ajustada	IC95%	p-valor
Eu tenho grande chance de pegar hepatite B (sim) — Suscetibilidade	1,73	1,30–2,29	<0,001
Se eu pegasse hepatite B, isso seria mais grave do que outras doenças (sim) — Gravidade	0,74	0,58–0,93	0,012
Vacinar-me contra hepatite B pode diminuir as chances de faltar ao trabalho (sim) — Benefícios	1,29	1,05–1,59	0,014
Vacinar-me contra hepatite B despenderia muito do meu tempo, pois são necessárias três doses (não) — Barreiras	1,41	1,11–1,79	0,004
Fico preocupado em ter uma reação à vacina da hepatite B (não) — Barreiras	1,43	1,14–1,80	0,002

RP: razões de prevalência; IC95%: intervalo de confiança de 95%.

Destaca-se que a perda de 7% de trabalhadores (n=32) nesta amostra, devido à falta de informações para as variáveis relacionadas à hepatite B, demonstrou similaridade com o grupo de trabalhadores respondentes com relação às características de sexo e idade. No grupo de perdas, 78,1% dos TS eram do sexo feminino e 75% tinham entre 21 e 49 anos; assim, minimiza-se a suscetibilidade do estudo a possível viés de seleção por não resposta.

## DISCUSSÃO

A prevalência de vacinação completa para hepatite B (autorrelato) entre TS neste estudo (56,9%) foi inferior àquelas constatadas em investigação no estado da Bahia em 2018, com TS da APS e MC (59,7%)^7^, e em inquérito prévio realizado com o mesmo grupo de TS no município de Santo Antônio de Jesus em 2015 (59,9%)^22^. Contudo, a prevalência de vacinação completa para hepatite B foi superior àquela encontrada em estudo com TS da Estratégia Saúde da Família em Montes Claros, no estado de Minas Gerais, em 2015 (52,5%)^6^.

Associaram-se à maior prevalência de vacinação completa para hepatite B: percepção de risco para a doença; gravidade em relação a outras doenças; percepção de que vacinação poderia reduzir absenteísmo; despreocupação em relação a tempo despendido para vacinar e à ocorrência de ESAVI.

A vacinação para hepatite B é importante estratégia de prevenção, pois 15 a 40% dos infectados desenvolvem insuficiência hepática, cirrose hepática e carcinoma hepatocelular, comprometendo vida produtiva na população afetada^11^. Investigação demonstrou que, em 2005, os custos médios por paciente, no setor público, variavam entre R$ 1.243,17 e R$ 22.022,61, a depender da complicação da hepatite B^23^. Após aproximadamente 20 anos, infere-se que esses gastos aumentaram consideravelmente.

A suscetibilidade percebida refere-se aos riscos subjetivos de adquirir determinada condição^16^. Assim, percepção de suscetibilidade varia conforme grau de risco ao qual um indivíduo se percebe exposto e se apresenta como condição determinante para adesão à vacinação em geral^8^. Sobre vacinação para hepatite B, há associação entre percepção de suscetibilidade e maior adesão à vacinação, a fim de evitar a infecção entre TS^7,22,24^.

Possivelmente, essa associação ocorre em virtude da exposição ocupacional, pois TS têm risco até três vezes maior de adquirir infecção por hepatite B em comparação à população geral^5^. Estima-se que o risco de contrair hepatite B após lesão com agulha pode chegar a 30%. Globalmente, a exposição ocupacional é responsável por aproximadamente 40% dos casos de hepatite B e C e por cerca de 66 mil infecções por hepatite B entre TS^25^. Neste estudo, TS que relataram grande chance de contrair a infecção por hepatite B apresentaram 70% de probabilidade a mais de se vacinarem quando comparados a TS sem essa percepção de risco.

Em 2012, investigação com TS do setor municipal de Belo Horizonte e inquérito com TS da APS e da MC em Santo Antônio de Jesus, na Bahia, em 2015, constataram que trabalhadores da área administrativa e de serviços gerais apresentaram menores taxas de vacinação quando comparados a médicos e enfermeiros^22,24^. Portanto, presume-se que trabalhadores administrativos e de serviços gerais não se percebem em risco em suas ocupações, em razão do contato menos frequente com o usuário do serviço. Entretanto, o risco de contato com material biológico ou superfícies contaminadas não é nulo; assim, a vacinação permanece como medida profilática mais eficaz e segura para prevenir infecção por hepatite B.

A gravidade percebida remete à possibilidade de a doença causar morte, reduzir funcionamento físico ou mental por longos períodos ou desabilitar permanentemente^14^. Neste estudo, TS que não compreendem a gravidade da hepatite B obtiveram maior prevalência de vacinação completa para a infecção. Relaciona-se esse cenário à maioria dos participantes deste estudo não ter graduação específica em cursos de saúde (apenas 30,9% eram trabalhadores assistenciais), nos quais disciplinas relacionadas a infecções proporcionam maior compreensão sobre a gravidade da hepatite B.

Em estudo com enfermeiras em dois hospitais no interior do estado de São Paulo, em 2012, 77,3% das participantes relataram receio de adquirir uma infecção grave, como hepatites B e C, e 92,5% consideravam-se suscetíveis a essas infecções^20^. A hepatite B é uma infecção grave, cujas repercussões oneram o sistema de saúde^23^.

A dimensão de benefícios caracteriza-se como crença na eficácia da ação recomendada para reduzir risco ou gravidade do impacto^17^. Neste estudo, maior prevalência de vacinação completa associou-se à redução nas chances de ausentar-se no trabalho. Em 2012, inquérito em dois hospitais paulistas, cujas participantes foram enfermeiras, obteve resultado similar: 100% das trabalhadoras entrevistadas afirmaram que o principal benefício ao se vacinarem contra hepatite B é evitar aquisição da infecção e, consequentemente, mitigar ocorrência de afastamentos do trabalho^20^.

Apesar da validade do benefício percebido para o TS, infere-se que seu pensamento se pauta sob uma óptica financeira, na qual se evita o absenteísmo, a fim de não haver riscos de desemprego. Então, a decisão desses TS de se vacinarem contra hepatite B não esteve atrelada à própria saúde, foi meramente atrelada à questão econômica.

As barreiras no MCS são ações efetivas para redução da ameaça da doença ou condição, contudo, elas podem ser consideradas inconvenientes, dispendiosas, pouco prazerosas ou dolorosas^15^. Neste estudo, intervalo de até seis meses entre as doses na vacinação para hepatite B não foi fator prejudicial à completude do esquema vacinal para a infecção. Investigação realizada entre TS de hospitais chineses evidenciou que intervalo entre as doses pode acarretar perda de oportunidade para vacinação, pois dos TS que não receberam as três doses da vacina, 40% alegaram que estavam muito ocupados para receber doses seguintes^12^.

Ademais, neste estudo, houve predomínio de TS da APS (77,7%), fator que pode promover vacinação para hepatite B, pois é rotina dos serviços nesse nível de atenção à saúde, como constatado em investigação anterior com a mesma população nesse município^13^. Enquanto TS da MC desempenham atividades pouco relacionadas à vacinação e estão distantes da sala de vacina, trabalhadores da APS se vacinam em seus próprios locais de trabalho e há estímulo para atualização dos cartões de vacinação.

TS que não relataram receio acerca da ocorrência de ESAVI alcançaram maior prevalência de vacinação para hepatite B, com probabilidade de 43% a mais de se vacinarem comparados a TS que referiram preocupação acerca de ESAVI. Provavelmente, esse cenário relaciona-se à compreensão acerca da baixa reatogenicidade da vacina para hepatite B: ESAVI são, comumente, leves e transitórios, enquanto graves são raros^1^, não sendo uma barreira para a vacinação. Em 2011, estudo transversal brasileiro realizado no estado de Minas Gerais constatou apenas 2,1% de incidência de ESAVI na vacina para hepatite B, sendo a quarta vacina com menor incidência^26^.

Em 2019, na Arábia Saudita, inquérito com trabalhadores assistenciais constatou que 31,2% dos TS não completaram o esquema vacinal para hepatite B e acreditavam que a vacina não é segura ou tinham dúvidas sobre sua segurança, enquanto apenas 8% dos trabalhadores da saúde que receberam as três doses recomendadas compartilhavam da mesma crença^11^.

Entre as limitações do estudo, há vieses de falsa resposta e recordatório, principalmente, quando TS foram questionados sobre vacinação para hepatite B^27^. A fim de minimizar vieses, solicitou-se cópia dos cartões de vacinação dos TS, estratégia ideal e recomendada para avaliação do desfecho de interesse^28^. No entanto, demonstrou-se que esses TS conhecem relativamente seus estados vacinais, portanto, considerou-se o autorrelato da vacinação para hepatite B para análise.

Apesar das limitações supracitadas, este estudo distingue-se por incluir não apenas profissionais de saúde, mas também as demais ocupações que trabalham no setor saúde. Logo, embora seja um estudo local, é factível que represente uma problemática existente em distintos lugares do país.

Este estudo evidencia que a taxa de vacinação para hepatite B dessa população está inferior à expectada e aponta como a percepção de risco e gravidade da doença pode potencializar aceitação de vacinas e completude de esquemas vacinais para hepatite B. Portanto, atividades de educação em serviço para TS devem incluir diálogo como estratégia de estímulo à vacinação por meio de abordagens que conscientizem acerca de eventos adversos esperados pós-vacinação e que a possibilidade de adoecer por infecção imunoprevenível, com destaque para a hepatite B, pode acarretar não apenas absenteísmo no trabalho, bem como hospitalização e óbito. Por fim, independentemente da formação ou função desempenhada, o TS deve assimilar as principais formas de transmissão da infecção e reconhecer a vacinação como ferramenta indispensável de prevenção.
